# Supplementary material for: The cost of doing nothing about a sleeper weed–Nassella neesiana in New Zealand
Source: PLoS One. 2023 Dec 27;18(12):e0295574. doi: 10.1371/journal.pone.0295574 (PMC10752537; doi:10.1371/journal.pone.0295574)
Supplement: Attachment — Submitted filename: Response to Reviewers.docx [file pone.0295574.s002.docx]

Reviewer #1: The paper highlights the potential for underinvestment in containment programs in countries with regional jurisdictions. In particular, if spread to another jurisdiction is ignored, there may be underinvestment. This relies on the untested assumption that spending more on containment could prevent geographic spread. Since this is probably a rare circumstance, the reliance on a strong assumption weakens the practical significance of the paper. Nonetheless, the issue of underinvestment in containment of invasive species in countries with regional governments has received little attention and should be raised. This is the main strong point of the paper. Its weak point is the lack of empirical support that the case study has broader significance. A second concern I have is that if the pest causes complete loss of access to infested paddocks, and if early paddock detection can prevent this, the model would have overstated the costs of allowing the species to spread. It is implicit in the paper that the species can be contained, which makes it important to address this concern in the revised manuscript. Subject to explicitly stating the strong assumption made about the capacity to prevent spread, and the cost-estimation assumption that infestation would go u8nimpeded until paddock access is lost, I recommend that the paper be accepted.

<We thank the reviewer for these helpful comments. We have addressed them in the new paragraph on Lines 295-314 in the track changes version of our resubmitted manuscript – copied below – and have made relevant changes to the final paragraph on Lines 315-323.>

<” Three key assumptions are fundamental to our model [23]. First, that the invasion of New Zealand by *N. nessiana* would continue unimpeded in the absence of regionally co-ordinated landscape-scale management programmes. Second, that to prevent livestock-mediated seed dispersal, and the animal welfare and product quality impacts of the seeds, invaded paddocks must be rested from grazing during the 3-month seeding period, resulting in a 25% p.a. loss in revenue. Third, that regionally coordinated management can prevent the spread. This assumption is supported by experiments in New Zealand showing low pasture reinfestation potentials from soil-borne seeds within a few years of repeated herbicide and soil cultivation treatments [15], and a propensity for slow spread in the absence of management [23]. The latter can be attributed to the predominantly short-distance dispersal of the seeds. The seeds clump together on the panicle at maturity due the intertwining of their hygroscopic awns and then fall to the ground at the base of the parent plant. Their long-distance dispersal is likely to occur only through human activities including the movement of seed-contaminated farm animals, forages, hay, soil, and river gravel, and of farm and roadside maintenance machinery [14]. In Australia, local eradication of *N. neesiana* has proven possible providing seeding is prevented using regular applications of herbicides such as fluproponate and glyphosate for 7-10 years (*pers. comm.* Steve Taylor, ACT Parks & Conservation Service, Australia). This confirms the predictions of the soil seed decay model developed in New Zealand [15]. Altogether, these ecological traits and management vulnerabilities indicate that preventing the geographic spread of *N. neesiana* in New Zealand is feasible.Regionally effective containment would depend on detection and sustained control efforts over many years [33]. The costs of these control methods would need to be estimated, shown to be equal to or less than the breakeven costs we have identified, and affordable.”>
